# Practical Strategies for Health Equity Researchers to Enhance Analytic Rigor and Generate Meaningful Insights From Qualitative Data

**DOI:** 10.5888/pcd19.220134

**Published:** 2022-11-03

**Authors:** Jennifer K. Felner, Vida Henderson

**Affiliations:** 1 San Diego State University School of Public Health, San Diego, California; 2 Institute for Behavioral and Community Health, San Diego State University Research Foundation, San Diego, California; 3 Fred Hutchinson Cancer Center, Public Health Sciences Division, Seattle, Washington; 4 University of Illinois at Chicago, School of Public Health, Division of Community Health Sciences, Chicago, Illinois

## Abstract

Researchers and public health practitioners increasingly need to leverage diverse methodologic approaches in health equity research that will lead to innovations in the assessment of health inequities and development of interventions to decrease health inequities. One well-suited approach is the use of robust qualitative methods (alone or in combination with quantitative methods). As more health equity researchers employ qualitative methods in their study designs, additional guidance is needed on how to conduct robust and rigorous qualitative data analyses. We share a 4-step analytic strategy for health equity researchers and practitioners — particularly those with limited training in qualitative data analysis — that can be used to effectively execute qualitative analysis to inform health equity–driven efforts. These strategies will guide those less experienced in qualitative methodology to employ a pragmatic approach to analysis that is sound, reasonable, and produces meaningful insight that can be used to inform efforts to advance health equity for communities with the greatest needs.

**Summary**

**What is already known on this topic?**

Integrating qualitative methods in study designs allows researchers to understand the relationships and contexts that influence health.

**What is added by this report?**

As more health equity researchers employ qualitative methods in their study designs, there is a need for additional pragmatic guidance on how to conduct robust and rigorous qualitative data analyses. We offer a 4-step strategy for analyzing qualitative data and discuss health equity implications for each strategy.

**What are the implications for public health practice?**

These strategies will guide those who are less experienced in qualitative methodology to use a pragmatic approach to analysis that is sound, reasonable, and produces meaningful insight.

## Introduction

Qualitative methods use nonnumerical or nonstatistical processes to explore human behavior and experiences in context as well as complex social-level and structural-level phenomena, including the social production of health (1–3). Because health equity–driven research prioritizes eliminating socially unjust differences in health such that all have equitable access to resources, quality health care, and opportunities to be healthy, qualitative methods are an important tool in the health equity researcher’s or practitioner’s toolbox. Employing qualitative methods (alone or in combination with quantitative methods) offers opportunities to produce new insights into the sources of health inequities (4–6) and leads to innovations in multilevel intervention development to decrease health inequities (1,5,7).

Major public health funding bodies encourage researchers to propose study designs that integrate qualitative and quantitative data (8). Integrating qualitative methods in study designs allows researchers to develop a more nuanced and holistic understanding of relationships and contexts that influence health than quantitative methods alone can (1,8). As more researchers and practitioners employ qualitative methods, there is a need for accessible and straightforward guidance on how to analyze and identify meaning within qualitative data, particularly among those without formal training in qualitative methods. Understanding qualitative data is especially needed within the context of health equity research, in which qualitative methods may be a primary source of information about how and why inequities exist and what people think should be done to advance health equity for their communities.

Considerable time and effort are required to develop expertise in qualitative analysis; however, time and resources may be limited for those working in health equity–focused research and public health practice. Therefore, we share an overview of a systematic, yet pragmatic, qualitative analysis approach to explore phenomena in context, elevate voices of those affected by health inequities, and inform health equity–focused interventions and related efforts. We will not delve into additional details on the use of qualitative methods for health equity research, assessment, and evaluation (for a recent review, see Shelton et al [5]). Our pragmatic process follows some of the analytic strategies of applied thematic analysis (9) and other approaches popular in the health sciences, such as constructivist grounded theory (10) and phenomenology (11). Definitions of key terms are provided in Table 1.

**Table 1:** Key Definitions for Qualitative Analysis Processes in Health Equity Research

Term or Concept	Definition
Code, coding, codable unit	Codes are key ideas in the form of a word or short phrase used to organize and categorize segments of data; codes provide a structure to identify main ideas and higher-level phenomena across the data (ie, themes). Codes are like sticky notes attached to important parts of data to be retrieved later. Note: codes are not the same as themes. Coding is the process of organizing the data by attaching codes to relevant segments of text to later retrieve that segment for identification of themes. A codable unit is a discrete segment of data or text to which codes are applied or attached.
Codebook	A codebook is a comprehensive compendium of codes (including code families and subcodes). A practical codebook will include code names and definitions, when to use or not use a code, and an example quote taken from data that illustrates application of the code.
Code family	Code families are sets of codes that share similar topics or ideas and are grouped together in the codebook
Code summary	A code summary is a data reduction technique that summarizes information conveyed by participants for each code or code family.
Computer-assisted qualitative data analysis software (CAQDAS)	CAQDAS uses computer-based software to assist in qualitative data management and coding processes. Examples include NVivo (QSR International), Maxqda (Verbi GmbH), Atlas.ti (Atlas.ti Scientific Software Development GmbH), and Dedoose (SocioCultural Research Consultants, LLC). It is not necessary to use CAQDAS to conduct sound qualitative data analysis, but the advanced tools available may be helpful and increase the speed of the analytic process.
Constructivist grounded theory	Constructivist grounded theory is a qualitative research approach that aims to develop new, midlevel theories to explain social phenomena or processes. The approach is inductive and iterative in nature, with each step in data collection and analysis informing the next. Researchers employing Constructivist Grounded Theory do not propose to be neutral observers, but rather acknowledge that data and theory development are co-constructed by both the researcher and participants.
Deductive code	Deductive codes are predetermined codes (identified before analysis); also referred to as a priori or index codes. Deductive codes tend to capture general ideas that lack the nuance of more specific ideas expressed in the data. These are often based on existing or working theories or conceptual models, prior literature, and research questions.
Descriptive qualitative analysis	Descriptive qualitative analysis aims to generate a comprehensive summary and overview of the data, focused on the who, what, and where of events. Researchers stay close to the data and do not necessarily analyze the data with the goal of identifying complex processes or theoretical understandings of phenomena.
Inductive code	Inductive codes are those that are not predetermined (a priori) and are grounded in the data (ie, the researcher[s] did not identify the codes before beginning the analysis process). These codes are typically identified through memoing, close reading of the data, or both. In vivo codes are a type of inductive code which use verbatim words or phrases from 1 or more participants.
Intercoder agreement	Intercoder agreement (ICA) is an assessment of how similarly (ie, reliably) 2 or more coders are applying codes to the data.
Interpretive qualitative analysis	Interpretive qualitative analysis aims to move beyond description to uncover more complex processes or phenomena, often with the broader goal of developing theoretical or conceptual models based on analysis.
Memo, memoing, and analytic memos	Memos are brief, written “notes to self” (a few words or sentences) used to capture initial impressions of the data and salient ideas; they are useful to immerse the researcher(s) in the data and to inform the development or identification of inductive codes. Memoing (the process of writing memos) is also referred to as “annotating” or “jotting in the margins.” Analytic memos capture ideas or reflections about the data, analytic choices, or revelations that occur during coding and other analytic procedures.
Percent agreement	Percent agreement is an approach to assessing ICA by calculating number of instances when coders agree (ie, apply codes the same way) divided by the number of instances of coding agreement and coding disagreement (number of codes in agreement divided by [number of codes in agreement plus number of codes in disagreement]); >80% agreement is often considered sufficient.
Phenomenology	Phenomenology is a qualitative approach that aims to identify the essence of a phenomenon or process. Phenomenology focuses on deeply understanding and elucidating the lived experiences of a group of participants with respect to a specific phenomenon or process.
Reflexive memo	Reflexive memos capture thoughts about one’s positionality, relationship to participants, biases, and power balances between researcher(s) and participants or the communities from which they come.
Statistical agreement	Statistical agreement is an approach to assessing ICA by calculating a statistic of coding agreement that accounts for chance; a Cohen kappa is a popular statistical approach, with >0.61 often considered sufficient.
Subcode	Subcodes are finer-grained concepts that are related to a higher-level code (sometimes referred to as child codes in contrast to higher-order parent codes).
Subjective agreement	Subjective agreement is a nonmathematical or statistical approach to assessing ICA in which coders simply compare and contrast their code applications across segments of text and identify instances where they have applied different codes.
Thematic analysis	Thematic analysis identifies and describes implicit and explicit ideas within and patterns across the data, that is, themes.
Theme	A theme is a cross-cutting, high-level concept that links ideas across data; the “a-ha,” “so-what,” or “big take-away” from the data; themes are more abstract than codes and are often identified from the coding process. Most analyses will yield multiple themes (ie, multiple “so-whats?”) and may also yield subthemes (a more fine-grained concept that is related to a specific element of a theme).

## Analytic Strategies and Health Equity Implications

We provide a set of analytic steps that we have each applied to multiple qualitative data sources, including data from semistructured and unstructured interviews and focus groups (eg, data in the form of audio files and verbatim transcripts), participant-observation and ethnography (eg, data in the form of field notes), narratives (eg, data in the form of written or published text), and photovoice (eg, data in the form of photos and oral or written analysis of photos). For simplicity, we will focus on analysis of verbatim transcripts herein. These steps can be applied using computer-assisted qualitative data analysis software (CAQDAS; eg, NVivo [QSR International], MAXQDA [VERBI GmbH], Atlas.ti [Atlas.ti Scientific Software Development GmbH], Dedoose [SocioCultural Research Consultants, LLC, 12]); or basic word-processing software and spreadsheets (13,14). A summary of each analysis step and the estimated timeline for completion are provided in Table 2.

**Table 2:** Summary of Analysis Steps and Estimated Timeline^a^

Analysis step	Key process	Estimated time for completion
0	Transcribe audio data verbatim Data organization (collate transcripts)	Usually takes 2–3 weeks for a set of transcripts to be returned from a professional transcription service; allow extra time for in-house transcription by members of the research team; allow extra time if transcripts need to be transcribed and then translated into another language
1	Memo subset of transcripts (or all transcripts, as relevant for analytic goals)	1–2 h per transcript (dependent on length and familiarity with data)
2	Compile and reduce memos Develop initial codebook (code families, subcodes, code definitions, criteria or directions for code application)	1–4 weeks (dependent on amount of data and number of memos and codes)
3	Access intercoder agreement Update or refine codebook	2–4 weeks (dependent on length of transcripts, number and skill or experience of coders, level of difficulty or ease of achieving desired percent agreement in coding)
3	Code all transcripts (continue to refine codebook if needed)	1–3 h per transcript for ~1 h of audio (speed will increase as coders become familiar with codebook)
4a	Export quotes by code, code frequencies, code co-occurrences, and any other visualization of interest (eg, code networks) Write code summaries of each code and code family Share code summaries with all team members	If 1 researcher or team member is writing code summaries: 2–3 weeks (dependent on amount of data and researcher skill) ~ 1–2 weeks to allow for team to read summaries and contribute themes
4b	Develop themes or overarching concepts Use code summaries to help explain each theme, as relevant Identify which themes address specific research or analytic goals, as relevant Refine themes through dialogue and writing	2–4 weeks (highly dependent on amount of data, complexity of analysis, researcher skill)
Beyond analysis	Write up or prepare presentation of results for dissemination	1–3 weeks (dependent on complexity of analysis, researcher skill, and dissemination outlet [eg, article vs presentation])

a Estimated timeline based on total of 20 hours of work per week. May vary depending on how much time is dedicated to each step and how many team members are working on certain aspects of analysis.

The analytic steps outlined herein are a team-based process. We firmly believe in involving diverse research teams in health equity research broadly and in analysis specifically. This means diversity in terms of methodologic or practical training, social identity or position (eg, race, gender, class), or research profession (ie, when possible and germane to the study goals, both professional researchers or public health practitioners and community partners are involved in analysis).

## Step 1: Memoing, Annotating, Jotting in the Margins

In the first step of the qualitative analysis process, team members write and apply analytic memos to the data, known as memoing (also referred to as annotating or jotting in the margins). Memos are brief “notes to self,” capturing initial impressions of the data and salient ideas that may be analytic or reflexive (15). They are usually a few words or sentences and can be directly attached to the data by physically writing notes in margins on hard copy or by using electronic track changes features in software to identify important or salient ideas or thoughts. Writing is an important element of qualitative analysis; writing memos allows researchers to begin immersing themselves in the data from the outset by formulating initial ideas and impressions in narrative form, and it is an initial step in understanding the depth and range of participants’ thoughts, ideas, and expressions (15,16). Additionally, writing memos ensures that subsequent code development (step 2) is grounded in the data.

In this step, each team member is randomly assigned 1 to 2 transcripts to memo. When timelines are very tight, research teams may elect to memo only a subset (eg, 15%–20% of transcripts) of randomly selected or purposefully selected transcripts for maximum variation across data or participant types. Ideally, all transcripts will be memoed by a member of the research team.

### Implications for health equity

Understanding lived experiences and root causes of health inequities requires deep exploration and inquiry into complex, multilevel factors that may affect multiple domains of a person’s or community’s health. Memoing helps the health equity researcher move beyond simply identifying and applying a priori or index codes (ie, predetermined codes) and enables researchers to be open to the direct lived experiences, thoughts, and ideas that are directly voiced or conveyed by participants. In addition, reflexive memoing can be used by health equity researchers as a process to reflect on their position regarding the research topic and communities of focus, relationship to participants, biases, and power balances that might affect the analysis process and findings generated.

## Step 2: Compile Annotations and Develop Codebook

After memoing, or a first pass of writing memos (some researchers memo throughout the analysis process, including during coding [step 3]), is complete, analytic memos can be compiled into a list to inform the identification of codes and development of the codebook. Word processing or CAQDAS can be helpful to easily output the memos into a single document. Reflexive memos may or may not be appropriate to include in this list, depending on the goals of the analysis. Once the memos are in a single document or list, a single researcher or multiple research team members read through the memos and reduce them to a few central words or short phrases that capture the essence of the memo. We recommend retaining a copy of the memos in their original form, which may be useful at later stages of analysis.

Research team members then read the reduced memos to identify key ideas and group them into “buckets” that are linked through a central idea. This process will inform the development of codes to organize and categorize segments of the data. Codes can be organized in a codebook, with each code represented by a descriptive word or phrase characterizing its meaning. Note that codes are not themes. Codes are simple, descriptive ideas. They are not higher-level concepts based on identified and interpreted patterns in the data. Codes are in service of identifying themes (16). Many researchers conflate these 2 concepts.

Depending on the goals and complexity of the analysis, codes may have a hierarchical structure in which codes are organized within code families of similar topics or ideas or into more fine-grained subcodes. The overall purpose of codes is to organize and categorize segments of data such that main ideas can be identified, interpreted, and shared (step 4b). A practical codebook will include names and definitions for each code and example quotes taken from the data that illustrate when codes should be applied. Often, details will include when to use and when not to use a code. The more detailed the codebook, the easier it will be for those applying codes to do so consistently and reliably.

Research team members should collaboratively develop codes and draft and refine the codebook (eg, clarify definitions, ensure codes are mutually exclusive, ensure code names are sufficiently descriptive). Codes directly informed by memos or reading of the data are referred to as inductive codes (ie, grounded in the data). However, researchers often have predetermined concepts they want to capture based on conceptual or theoretical frameworks, interview or focus group questions, prior research, or research questions or study aims. Codes based on these predetermined concepts are referred to as deductive codes, a priori codes, or index codes and tend to capture more general ideas than inductive codes. Most codebooks will include both inductive and deductive codes.

As with all analytic phases, openness to multiple iterations for refinement is needed. Another consideration among research teams is the level of coding that is needed for a given project. Although there is no predetermined number of codes appropriate for any given project, teams must decide if the analysis requires macro-level coding (codes that capture broader characterizations) or more detailed and specific codes or subcodes.

### Implications for health equity

This step is an opportunity to leverage existing health equity–related frameworks, theories, or models to identify additional codes or code families and to guide the subsequent analytic processes. If researchers aim to understand how a certain health equity–related theory applies to or is aligned with the data, they might use constructs of that theory as codes (or to frame or categorize themes [step 4]). By using in vivo codes (a type of inductive code that use verbatim words or phrases from the data), however, the analysis is grounded in participants’ perspectives and retains their original words and language. Additionally, code development may be an initial step to inform new theory development or refinement when existing theories do not adequately capture relationships found in the data. For example, individual-level health behavior theories are often insufficient when examining a research question with an equity lens. Inductive coding can help researchers uncover multilevel factors that contribute to a person’s ability to enact behavioral change, resulting in theoretical frameworks that consider social and other external factors that affect equitable outcomes.

## Step 3: Coding Data

Coding is the process of organizing data by attaching codes to relevant segments of text We liken this to placing a sticky note on parts of the transcript to flag it for later retrieval. Transcripts (and other documents, such as photos) can be coded with CAQDAS or word processing software and spreadsheets (13,14). Once the data are coded, researcher(s) can then retrieve and review the coded text segments to identify the higher-level concepts across the data (themes). Research teams typically have at least 2 people, referred to as “coders,” who code transcripts, especially when there is a large amount of data.

### Selecting text segments or codable units

An important consideration before coding is to determine what will be considered a codable unit. A codable unit is a discrete segment of text to which codes are applied. A common coding misstep is inappropriate determination of a codable unit (or a lack of training for the coders on what to code). When coding text, a codable unit must make sense when standing alone. It is often unhelpful for coders to select a few words or even a single sentence that does not encapsulate meaningful context as a codable unit because it will be difficult to interpret when reducing data (step 4a) and identifying themes (step 4b). For example, the research team may decide that a complete thought is considered a codable unit (which could be a few sentences or paragraphs) or that an entire response to each interview question is a codable unit.

### Coding reliability

Before coding data independently, it is common for 2 (or more) coders to both code approximately 10% to 25% of the data to assess how similarly they are applying codes; this is referred to as intercoder agreement (ICA). If there is insufficient ICA, which means that coders are applying different codes to the same segments of text, there may be codebook issues to be addressed, such as unclear code definitions, missing codes, overlapping or redundant codes, or a need for more training. Once sufficient ICA is reached, coders may begin coding the remaining data independently. Best practice is for an experienced research team member to preselect codable units for the coders during ICA assessment (eg, by highlighting codable units before applying any codes). This will help coders learn what is considered a codable unit for the particular analysis and make it easier to assess coding reliability, because coders will each be working from the same point of reference (as opposed to potentially selecting and coding different segments of text).

Generally, there are 3 approaches to assessing ICA: subjective agreement, percent agreement, and statistical agreement, with debate about which, if any, is the most appropriate to use in qualitative data analysis (9,17,18) (for a useful overview of the debate, see O’Connor and Joffe [19]). Overall, the selected approach to assessing ICA will be driven by project goals, research team skill and access to analytic resources, philosophical underpinnings of the study, and feasibility — each of which may vary by study even if conducted by the same research team. We believe the goal of assessing ICA should be to generate research team dialogue and reflection that will inform codebook improvements and increase the coders’ confidence and effectiveness in coding important segments of the data. This assessment should be considered a helpful process, rather than an end goal to “prove” the reliability of an analysis and subsequent findings.

To assess subjective agreement, coders simply compare and contrast their code applications across segments of text and identify instances of differing code applications. Discussion is used to determine which, if any, code application is right, then coders make adjustments to the codebook or their coding as needed (sometimes referred to as consensus coding). Mathematical calculations are not conducted in this assessment of ICA.

To assess percent agreement, a research team member tallies the number of instances in which coders applied the same code(s) to preselected segments of text. That number is divided by the total number of instances in which coders applied the same code(s) to preselected segments of text plus the number of instances in which coders applied different code(s) to preselected segments of text. 

Statistical agreement extends percent agreement by calculating a statistic of code agreement accounting for chance. Some suggest statistical agreement is superior to percent agreement because it accounts for chance and as such, should be prioritized to assess coding reliability (19,20). However, we do not ascribe to this notion for every study or research team. The most commonly used statistic is Cohen kappa (κ) and, more recently, Krippendorff alpha (α) (19). These statistics can be calculated by using multiple CAQDAS software programs as well as online calculators. For a free, detailed resource describing how to calculate and use these statistics, see Geisler and Swarts, chapter 5 (17). For a detailed application in applied public health research, see MacPhail et al (21).

### Pilot or first round assessment of ICA

As described above, codable units should be the same and be preselected for coders. For first round ICA, 2 or more coders code an entire transcript or only half of a transcript — this is largely dependent on the amount of data. We suggest that when there are fewer than 20 transcripts, the coders may code half of a single transcript during this first round. The research team should predetermine the acceptable minimum standard for reliable ICA; 80% for percent agreement (scale of 0%–100%) and 0.61 for statistical agreement (scale of 0–1) have been identified as common minimum standards, although there is a lack of consensus on these standards (19). On the basis of this minimum standard, the research team can determine if coding is insufficiently reliable and thus codebook updates are needed (they almost always are) or if additional coder training is needed.

### Second round assessment of ICA

Once the codebook is refined based on pilot or first round assessment of ICA, coders should code another full transcript (or portion of a transcript), recalculate ICA, and again discuss and implement any needed changes to the codebook in partnership with the broader research team. This process is typically repeated until sufficient reliability is achieved. Notably, sufficient ICA may be more difficult to reach with codebooks that contain a large number of codes and subcodes. However, that is not a reason not to include all necessary codes in a codebook. Coders should predetermine the code level for which they will determine agreement (eg, code family, subcode). Once sufficient ICA is reached, the remaining transcripts can be divided among the coders.

### Implications for health equity

Journal reviewers or researchers who are less familiar with qualitative methods tend to rely excessively on the utility of ICA as an attempt to lend quantitative credence to qualitative methodology. Although assessing coding reliability is a useful analytic process that offers the opportunity for refinement to ensure that coding processes and the meaning of codes are valid, it is more important as an opportunity to engage in additional dialogue and reflection that can ensure a health equity stance in the analysis process.

Coding requires deep and focused attention to the data, which enables thorough insights, facilitates validity and transparency in interpretation of findings, is a vehicle for understanding participants’ perspectives and identifying and discovering relationships, structures the data, and makes it accessible (22). All of these attributes are critical when seeking to understand the complex interplay of factors that affect health equity. Codes are important guideposts for team members as they discuss, distill, and seek to understand data during the analysis process.

## Step 4: Data Reduction and Theme Identification

### Step 4a: data reduction

Data reduction is a purely descriptive phase of the analysis process. Data reduction is taking a large amount of data (all data excerpts categorized by code) and distilling it to key distinct points that were conveyed by participants. To achieve this, the next step in the analytic process is to organize or group all coded text segments (ie, excerpts) by each respective code. CAQDAS or other specialized software allows researchers to easily export all coded segments for each code in desired formats (eg, Word, Excel, PDFs). One strategy for reducing data is for 1 or 2 team members to write data summaries for each code or code family by reading excerpts for each code from exported documents and narratively summarizing what was expressed by participants for each code or code family. This will result in data reduction, not in themes. Once summaries are completed, all team members read code summaries and collectively contribute thoughts and ideas for salient themes derived from the data. For quantitative data-oriented researchers, codes may be thought of as variables, excerpts as raw data, and summaries as descriptive results.

We recommend that researchers do not attempt to identify themes during the data reduction phase, although of course, some ideas will begin to form. This phase is only about reducing data before developing themes. Team members should have a thorough understanding of what was expressed by participants, independent of any given team member’s thoughts about relationships and associations. This allows each team member to reflexively contribute their own thoughts and ideas related to concepts expressed and sets the stage for increased depth and range of ideas during the theme-generation phase. As with most phases of qualitative data analysis, summarizing results is iterative. For example, after examining initial written summaries, teams may decide that it is necessary to conduct additional data coding to get more granular details of a particular code or code family, or different research questions may require additional examination of a particular phenomenon.

### Step 4b: theme generation and meaning-making

At this point, analysis moves from categorization to theme generation and meaning-making. Two key types of qualitative analysis goals should be considered in preparation for this step. The first is descriptive qualitative analysis, which aims to identify and detail the who, what, and where of events. In these analyses, researchers stay close to the data and do not aim to uncover processes or phenomena that are under the surface of the data or develop theoretical or conceptual models based on the data (23). The second is interpretive qualitative analysis, which aims to move beyond description of the data to uncover more complex processes or phenomena, often with the broader goals of developing or informing theoretical or conceptual models and answering research questions. Both descriptive and interpretive analytic goals are often applied to the same set of data; however, it is recommended that researchers identify the goals of their study well before analysis begins to determine whether goals of analysis are descriptive, interpretive, or both. Qualitative health equity research and analysis are often interpretive in nature, given the common goal of identifying root causes of health inequities.

Regardless of the analytic goal (descriptive or interpretive), moving from codes to themes is perhaps the most abstract and time-consuming phase of the analysis process. Sometimes researchers get bogged down with ensuring near perfect ICA when that energy and time is better spent on data interpretation and theme generation. Themes are high-level concepts based on patterns and linkages in the data — representing shared units of meaning connected by a central organizing concept or phenomenon (24,25). We conceptualize themes as the “a-ha,” “so-what,” or “big take-away” of the data. Clarke and Braun (24) explain that themes differ from basic topic summaries of the data in that “themes [are akin to] key characters in the story we are telling about the data (rather than collection pots into in which we place everything that was said about a particular data domain)” (p. 108). Even a descriptive qualitative analysis should strive to move beyond simply reducing the data and grouping data into buckets (step 4a) to identifying higher-level themes across the data.

So how do researchers identify and detail the themes of their data? Strategies have been described in prior publications (16,26–28). Some key strategies involve identifying 1) repetitions across the data, though repetitions alone are insufficient to signify a theme; 2) metaphors and analogies in the data (this could be found in both the textual, coded data as well as in the analytic memos developed during step 1); 3) transitions in the data (ie, natural or intentional shifts in participants’ comments or words that connect ideas or concepts such as “because,” “since,” “if,” or “then”); 4) similarities and differences across the data or multiple sources of data (ie, how a described experience or perspective is similar and different across transcripts or across data from various sources such as interviews, focus groups, or observations); 5) missing data or “silences in the data” (ie, considering what was left unsaid or not mentioned in and across the data may shed light on topics that participants wish to avoid or that researchers may have thought would be relevant but were in fact not relevant for the participants); and 6) elements of or connections to established theory, which may help place the findings in a broader conceptual or theoretical context (9,26). In addition, it may be helpful to develop thematic networks or maps to visually connect ideas between higher-level organizing themes and more concrete ideas related to the theme (for examples, see Attride-Stirling [29] and Richards et al [30]). Some CAQDAS produce visualizations of relationships between codes or patterns in the data; however, simply drawing these networks or maps by hand is effective. Contrary to some methodologic discourse, we suggest themes do not “emerge” during qualitative data analysis (although we have each been guilty of using this language in the past), but rather are “*produced* by the researcher through their systematic analytic engagement with the dataset, and all they bring to the data” (18, p. 9) on the basis of their own experiences, personal identities and social positions, and training.

In our experience, the most helpful theme-generation process involves some or all of the steps described here plus multiple rounds of research team dialogue based on the coded data and code summaries in the context of the study aim(s). In this approach, research team members apply their own theoretical lenses and knowledge to the reduced data to discuss and identify themes. Moving from summarizing the data to identifying themes takes time, intellectual work, and makes some team members uncomfortable because it requires conceptual leaps that transform lived experiences to higher order concepts. However, just as we make conceptual leaps in quantitative analysis, the same is true for qualitative analysis.

Salient ideas are not necessarily the most commonly occurring; therefore, avoid equating frequency with importance. Ideas expressed by only 1 participant may be as important as ideas expressed by multiple participants. Likewise, a few participants may have discussed a particular idea in depth, resulting in a high frequency of a specific code, but that frequency of code may not indicate a meaningful high-level pattern or phenomenon. Some researchers working with qualitative data may choose to use counting or numbers when relevant for their analytic goals and audience, or when frequency has theoretical or practical meaning (31), but we suggest this be used carefully and sparingly.

### Implications for health equity

Perhaps the most important function of qualitative research for the health equity researcher is the opportunity to elucidate and contextualize lived experiences and social processes to inform intervention and program development, policy, evaluation, and theory. Those affected by health inequities are often prey to underrepresentation; a lack of understanding about their experiences; and the social structures, norms, and ideologies that perpetuate health inequities. Data derived from qualitative methods must accurately and appropriately describe conveyed experiences, and interpretations and implications of data must be thoroughly examined and considered among diverse research teams (eg, by discipline, social identity, training).

An important opportunity to apply the analytic processes we have outlined is within the context of community-based participatory research (CBPR) projects. CBPR has the potential to link research and action to advance health equity by authentically and equitably involving community partners in all aspects of the research process (32,33) (for examples of participatory qualitative data analyses, see Dill [34], Hebert-Beirne et al [35], and Switzer and Flicker [36]). Care should be taken to determine the extent to which community partners wish to engage in each step of the analytic process. Such involvement of community partners has the potential to ensure that findings are sufficiently grounded in the needs, ideas, and experiences of those affected by health inequities and that recommendations adequately reflect community priorities. At a minimum, if the analysis process itself is not participatory within a CBPR project, it should be done with “accountability to the community” (37, p. 851), such as sharing preliminary findings (often referred to as “member-checking” [38]) with community partners or other stakeholders to validate and offer additional considerations regarding researchers’ interpretations and recommendations to advance health equity through intervention development or policy making.

## Beyond Analysis: Reporting Findings

Qualitative data analysis is iterative in nature, and the multiple steps involved, even if nonlinear, should be thoroughly described in publications and presentations of findings (including processes such as memoing, codebook development, testing and refinement, and approaches to theme generation) (39). Typically, researchers report findings by theme, including description and interpretation of the theme, and use verbatim excerpts (quotes) from the data to provide evidence for the theme and honor participants’ voices. Quotes should be edited only for clarity (it should be clearly noted when an excerpt has been edited) and must stay close to participants’ original words or phrases, because it is inappropriate to correct grammar or change a participant’s words. We caution against using too many quotes to support a theme, as a high volume of verbatim text can be cumbersome for a reader to digest — it is the researchers’ job to explain the theme for the reader, not the reader’s job to discern the underlying meaning of multiple quotes. For a resource on how to report findings for dissemination to various audiences, see Guest et al, chapter 10 (9); for a how-to on writing thematic statements to enhance presentation and translation of findings for public health and health sciences audiences, see Sandelowski and Leeman (27).

## Applications of Analytic Process in Health Equity Research

The steps we have laid out are a foundation for a meaningful yet pragmatic analytic process, rather than a strict recipe for how to analyze qualitative data within the context of health equity research. Indeed, every project has different goals; thus, the application of these steps may vary considerably between projects, even those led by the same team of researchers or practitioners. In the Box, we provide brief examples of how this broad analysis process was applied to 2 studies focused on elucidating the determinants of and identifying solutions to health inequities affecting 2 different communities.

**Box. Application of Analytic Process in Health Equity Research**

**In Exploratory Research**

In 2017, J.K.F. led a community-based participatory research (CBPR) study in partnership with a group of young adult co-researchers to examine the experience of low-income young adults of color (various races and ethnicities, predominantly Black and Latinx) aging out of lesbian, gay, bisexual, transgender, queer, or questioning (LGBTQ) social services for youths (40,41). Our collaborative research team gathered multiple sources of qualitative data, including focus groups with youths and analyzed data by using an adaptation of analysis steps 1 through 4. This adaptation ensured the young adult co-researchers could actively participate in analysis by removing barriers to participation, such as lack of computer access or experience with computer-assisted qualitative data analysis software (CAQDAS). In turn, the research team could produce findings and recommendations with local validity.

Memoing verbatim transcripts (step 1) was neither appealing nor accessible to our collaborative research team, and as such might have alienated the young adult co-researchers from the analysis process. Instead, we listened to audio recordings of the focus groups and wrote notes about what we each found useful to answer the study’s research questions between group meetings.

Then in collaborative analysis meetings, J.K.F. played preselected segments of focus group recordings most germane to the analytic goals of the study and asked the young adult co-researchers to verbally respond to the following questions: “What big ideas do we hear in this clip? What words or phrase might we use to categorize what participants are discussing?” (41, p. 116) — akin to verbal memos. These became the basis for an initial set of predominately inductive codes and definitions (step 2). After multiple rounds of discussion and code edits and adaptations, we manually applied codes to copies of the transcripts by highlighting text segments and writing in the margins (step 3), making coding decisions and iteratively editing the codebook as needed in real time — akin to subjective agreement (9). J.K.F. then applied the codes to the transcripts in CAQDAS Dedoose [SocioCultural Research Consultants, LLC]). As a group, we reviewed hard copies of coded excerpts exported from Dedoose, narratively summarized key ideas for each code (step 4a), and used a thematic network approach to visually document connections between codes and identify the “so-whats” of the data (themes, step 4b).

We presented preliminary findings to the community, including clients and service providers at LGBTQ-serving organizations, local groups of youth leaders, and other researchers, in multiple settings and used their feedback to finalize themes and make recommendations. This process facilitated community participation in data analysis to inform actionable solutions to advance health equity for low-income adolescents and young adults of various races and ethnicities, predominately Black and Latinx, aging out of LGBTQ social services for youths.

**In Intervention Development**

Black women at risk for inherited genetic mutations that increase their chances of getting breast cancer are only half as likely to receive genetic counseling and testing as non-Hispanic White women, yet Black women are 41% more likely to die from breast cancer (42,43). V.H. and a research team developed a culturally responsive narrative intervention video for Black women with hereditary risk for breast cancer to facilitate decision making about genetic counseling attendance (44).

To inform content and development of the intervention, our research team recruited Black women with a family history of breast cancer from a previous study to participate in one-on-one qualitative interviews regarding personal beliefs and experiences related to breast cancer and breast cancer risk and participate in story circles regarding community and family-related experiences and beliefs about cancer. To analyze the data, our team developed deductive codes based on the Integrative Model of Behavioral Prediction (45) and inductive codes based on our team’s analytic memos. After coding the data, our team reduced it by narratively summarizing coded excerpts and creating various data displays (matrices, networks, charts) that mapped onto our theoretical framework.

Themes from interviews and story circles were triangulated to detect commonalities, contradictions, and expansions. Themes from lived experiences and direct quotes shared during interviews and story circles were used to create the storyline, messaging, and educational content of the intervention video script. Our research team then conducted a series of multiple focus groups with additional cohorts of Black women with a family history of breast cancer, health care providers, and representatives from community-based organizations to get iterative feedback on scripts, storyboards, visual style and images, and the final video. Our team analyzed these data by using the same approaches as used for the interview and story circle data. The collection and analysis of these qualitative data resulted in an intervention that was culturally informed, responsive, and representative of Black women with increased breast cancer risk. This strategy can be applied to intervention development of decision aids that are aimed at mitigating inequities among any marginalized communities.

## Conclusion

We have shared strategies that can be used to effectively conduct qualitative analysis and generate meaningful results to inform health equity–related efforts. These strategies may be particularly useful for less-experienced health equity researchers and practitioners. Participants in health equity–focused qualitative and mixed methods studies give of their time and energy, often sharing intimate details of their needs, perceptions, experiences, and even fears. It is up to us as health equity researchers to honor these precious data by analyzing them thoroughly and with care.

As a final note, we invite readers to consider that qualitative methods in and of themselves are not aligned with the goals of health equity research. The research worldview and approach to knowledge generation of the researcher(s) and the practical goals of the research are more important than the methods used when it comes to advancing health equity through research (46). Thus, a health equity–focused research project should begin with a goal aligned with a health equity stance, such as identifying the roots of health inequities, facilitating the voices of communities affected by health inequities, or intervening on the socio-structural determinants of inequities. The selection of methods (qualitative or otherwise) and analytic strategies can then flow from said goal.
